# The correlation between NEDD4L and HIF-1α levels as a gastric cancer prognostic marker

**DOI:** 10.7150/ijms.34646

**Published:** 2019-10-21

**Authors:** Xingwang Jiang, Shangxin Zhang, Zihuan Yin, Yi Sheng, Qiang Yan, Ruochuan Sun, Mingdian Lu, Zhen Zhang, Yongxiang Li

**Affiliations:** 1 Department of General Surgery, First Affiliated Hospital of Anhui Medical University, Hefei 230022, People's Republic of China; 2 Department of Thoracic Surgery, Anhui chest hospital, Hefei 230022, People's Republic of China.

**Keywords:** gastric cancer, NEDD4L, HIF-1α, prognosis, co-expression

## Abstract

NEDD4L (neural precursor cell expressed developmentally down-regulated 4-like) protein is a member of ubiquitin ligases Nedd4 family. Although studies have shown that Nedd4L may act as a tumor suppressor in various cancers, including gastric cancer (GC), its clinical significance and the diagnostic value in GC is not well defined. HIF-1α (hypoxia inducible factor family of transcription factors) is actively involved in the metabolism of many tumors, although the relationship between its expression levels and clinical significance in GC still need to be established. In this study, the level of HIF-1α and NEDD4L mRNA and protein in 25 freshly frozen GC- and matched normal-tissues were determined by western blot and quantitative PCR (qPCR). Additionally, immunohistochemistry assay was performed to measure the protein level of NEDD4L and HIF-1α in 124 GC and 25 normal control tissues. We observed that the NEDD4L mRNA and protein levels decreased significantly (P < 0.001) in GC tissues, while that of HIF-1α increased (P < 0.001), and they both were associated with a poor prognosis, as was the case in patients with lower NEDD4L and higher HIF-1α expression (P < 0.001). On correlation analysis, a significantly negative relationship (r = 0.288, P < 0.01) was revealed between NEDD4L and HIF-1α expressions. Multivariate analysis revealed that co-expression of NEDD4L (P < 0.05) and HIF-1α (P < 0.001) were independent predictors of GC prognosis. Thus, the correlation of NEDD4L and HIF-1α levels may act as a prognostic marker of GC.

## Introduction

Throughout the world, a major cause of deaths due to cancer is because of gastric cancer (GC), amounting to 20% of the total world population leading to a huge burden on health and economy^1^. In 2012 alone, nearly 720 000 patients lost their lives due to gastric cancer and, more than 950 000 new diagnoses were made each year 2. The occurrence and development of GC is complex, multi-factorial, and involves several genes. In several cases, the disease is inoperable during diagnosis or may recur even after curative resection. Therefore, it becomes essential to determine a biomarker that is prognostic for the development and progression of GC, to enable an improved and accurate prediction of its recurrence.

Over recent years, NEDD4L has been shown to play key roles in the prognosis of tumors such as in prostate cancer^3^, non-small cell lung cancer (NSCLC)^4^, cancer of gallbladder^5^, gastric cancer 6, malignant glioma^7^, and colorectal cancer^8^. However, NEDD4L has been observed to have contradictory roles, as it promotes cancer of gallbladder cancer and malignant glioma, and has a protective role in colorectal and gastric cancer. NEDD4L is closest in homology to and shares similar E2 specificities with NEDD4, an evolutionarily conserved E3 ligase and a prototype protein like the ubiquitin ligases from NEDD4 family. It was first identified as a down-regulated gene in the course of development of the central nervous system of an early embryo. The members of NEDD4 family have a distinct modular domain architecture with domain for Ca2+ phospholipid binding (C2) at the amino terminal, the 2-4 WW domains for protein to protein interactions and the HECT domain at the carboxyl terminus^9^. The role of NEDD4L in the kidney as the inhibitor of the epithelial sodium channel (ENaC) is well-known^10^. NEDD4L also regulates the PI3K-AKT signaling pathway via PIK3CA ubiquitination. Although the NEDD4L expression has been evaluated in the prognosis of GC, a more elaborate study on a larger sample size is required to highlight its specific mechanism and clinical significance.

HIF-1 (hypoxia inducible factor), a transcription factor, is a heterodimer of the HIF-1α and HIF-1β subunits^11^ and has a key role in tumor cells for energy production for maintaining their metabolism 12. Normally, HIF-1α undergoes quick, proteasome-mediated degradation, but under hypoxic conditions, it is stabilized. It is overexpressed in various cancers, including that of the ovary, breast, uterus, cervix, and oropharynx and its overexpression often positively leads to poor prognosis^13^-^16^. HIF-1a is favorably linked to phosphorylated AKT. Studies have also shown that promotion of the AKT-HIF-1α-VEGF pathway, independent of hypoxia, aids in GC tumorigenesis and angiogenesis 17. While enhanced expression of HIF1α is often linked to poor prognosis in GC 18, nevertheless, some conflicting prognostic data is still reported 19, 20.

In GC, the PI3K-AKT-mTOR pathway may activate HIF-1α 17. Interestingly, NEDD4L regulates the PI3K-AKT signaling pathway via ubiquitination of PIK3CA 21. We hypothesized that the NEDD4L and HIF-1α co-expression plays an important part in clinical prognosis in GC. Therefore, in this study, we explored the HIF-1α and NEDD4L protein expressions in on a larger number of human GC tissues, their correlation and effect on the GC patient prognosis.

## Materials and Methods

### Extraction of total RNA and quantitative real-time PCR

Fresh frozen human GC tissue were used for extraction of total RNA using TRIzol Reagent from Invitrogen (USA), and reverse transcription was carried out using the ReverTra Ace qPCR RT Kit from Toyobo (Japan) as per instructions of the manufacturer. The NEDD4L and HIF-1α mRNA levels were determined by RT-PCR on the Sequence Detection System ABI 7900HT from Applied Biosystem (USA) and using SYBR-Green dye from Toyobo (Japan), and the following primers: NEDD4L, sense (5′- TCCAATGGTCCTCAGCTGTTTA -3′) and antisense (5′-ATTTTCCACGGCCATGAGA-3′); HIF-1α, sense (5′-ATCCATGTGACCATGAGGAAATG-3′) and antisense (5′-TCGGCTAGTTAGGGTACACTTC-3′); GAPDH, sense (5′-ATCAAGAAGGTGGTGAAGCAGG-3′), antisense (5′-CGTCAAAGGTGGAGGAGTGG-3′). The 2^-∆∆CT^ method was used to estimate the fold changes in expression.

### Clinical specimens

The department of general surgery in the First Affiliated Hospital of Anhui Medical University (Hefei, China) provided us with human GC tissue microarrays (TMA) of 124 primary GC and 25 normal gastric tissues that were randomly selected were obtained from between December 2006 and September 2008 and the follow-up period was from 2006 to 2013. No prior radio-, chemo-, or other tumor-specific therapy was given to any of the patients before the sampling. The time from the day 1 post-operation to the day of death was defined as the overall survival time (OS). Samples were collected afresh from 5 cm from the edge of the tumor and from corresponding normal tissues, dipped in liquid nitrogen for quick freeze, and kept at -80°C until RNA and protein were extracted. Two experienced pathologists examined the result of immunohistochemical staining. For TNM staging, the standard was the seventh edition of TNM Grading Standard specified by the American Joint Committee on Cancer (AJCC). All patients signed a written and informed consent form. The Institute Research Ethics Committee of Anhui Medical University's First Affiliated Hospital reviewed and approved this protocol.

### Western blotting

The RIPA buffer containing a mixture of protease inhibitor was added to the fresh-frozen tissues to extract total protein and their quantification was done using BCA Protein Assay Kit from Beyotime (China). Protein from the samples were separated on SDS polyacrylamide gel (10%) by electrophoresis and transferred to nitrocellulose membrane from Millipore (USA). After blocking for 1 h with nonfat milk (5% in tris-buffered saline with tween-20) solution at room temperature, anti-NEDD4L rabbit antibody (1:1000; Proteintech, China) and anti-HIF-1Α rabbit antibody (1:1000; Abcam, USA) were added to the membrane and kept overnight at 4°C. Then, the respective secondary antibody (1:10000, Proteintech, China) were added to the membrane and after 1h incubation at room temperature, enhanced chemiluminescence system was used to detect the immunoreactive signals. The internal control in this assay was GAPDH.

### Immunohistochemical staining and evaluation

A conventional immunohistochemical (IHC) staining protocol was used. Tissues from GC and healthy samples were fixed in formalin-fixed, paraffin- embedded, and used to prepare TMA. The TMA section was kept in xylene for removing paraffin, rehydrated in alcohol at different concentrations, then treated with 3% H2O2 and subsequently microwaved in the citrate buffer (10 mM, pH 6.0) for 5 min at 120 °C. The sections were blocked with BSA (bovine serum albumin, 1%) for 0.5 h, incubated overnight with anti-NEDD4L antibody (1: 100; proteintech), anti-HIF-1α (1: 300; Abcam) at 4 °C, and then with a secondary antibody labeled with peroxidase. The slides were then rated to assess the level of protein expression on the basis of the intensity of staining of the product. Scores were defined as: 0, no staining; 1, weak staining; 2, moderate staining; 3, strong staining) and percent of positive tumor cells were scored as: 0, none; 1, 1%-25%; 2, 25%-75%; 3, > 75%. The range of the final score was 0 and 9, defined as negative or weak (-, 0 ~ 2), and positive (+, 3 ~ 9).

### Statistical analysis

The software SPSS 17.0 (SPSS Inc., USA) and GraphPad Prism 5 (San Diego, CA) were used for statistical analyses. For analyses of NEDD4L and HIF-1α levels and clinicopathological factors, the Chi-square test was applied. The correlation between NEDD4L and HIF-1α were done by Spearman's rank test. The dissimilarities between gastric and non-tumor tissues in terms of the levels of HIF-1α and NEDD4L were compared using Student's t-test (two-tailed). For univariate and multivariate analysis, the model of Cox's proportional hazards was used to spot independent prognostic factors. The statistically significant values were P < 0.05.

## Results

### NEDD4L and HIF-1α expressions in fresh GC tissue

**Figure 1:**
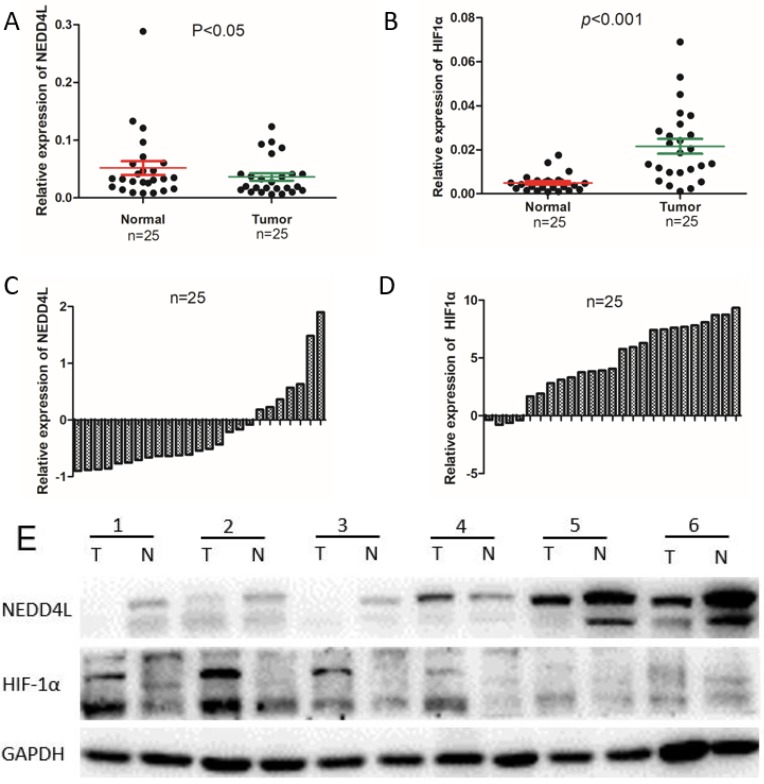
The mRNA and protein expression level of NEDD4L and HIF1α in clinical samples. Scatter plots of the relative expression of NEDD4L (A) and HIF-1α (B) mRNA in tumor and normal tissues. Bar plots of NEDD4L (C) and HIF-1α (D) expression in GC tissues compared with corresponding normal tissues. In each patient was presented as the log2 ratio of tumor tissue/normal tissue. (E) The protein expression level of NEDD4L and HIF-1α were analyzed by western blot assay.Representative protein expression level of NEDD4L and HIF-1α in 6 pairs of tumor (T) and corresponding normal tissues (N).

The qPCR was carried out to evaluate the NEDD4L and HIF-1α mRNA expressions in fresh GC- and corresponding normal-tissues. As per the Figure 1, the expressions of NEDD4L in GC tissues was notably down-regulated, whereas that of HIF-1α was upregulated. We carried out western blot to examine the association between levels of NEDD4L and HIF-1α in relation to their respective mRNA levels, and found that while NEDD4L expression reduced distinctly in GC tissues, the HIF-1α increased notably, which is in accordance with the qPCR result (Figure 1).

### Immunohistochemical staining for NEDD4L and HIF-1α levels in matched GC and normal tissues

**Figure 2:**
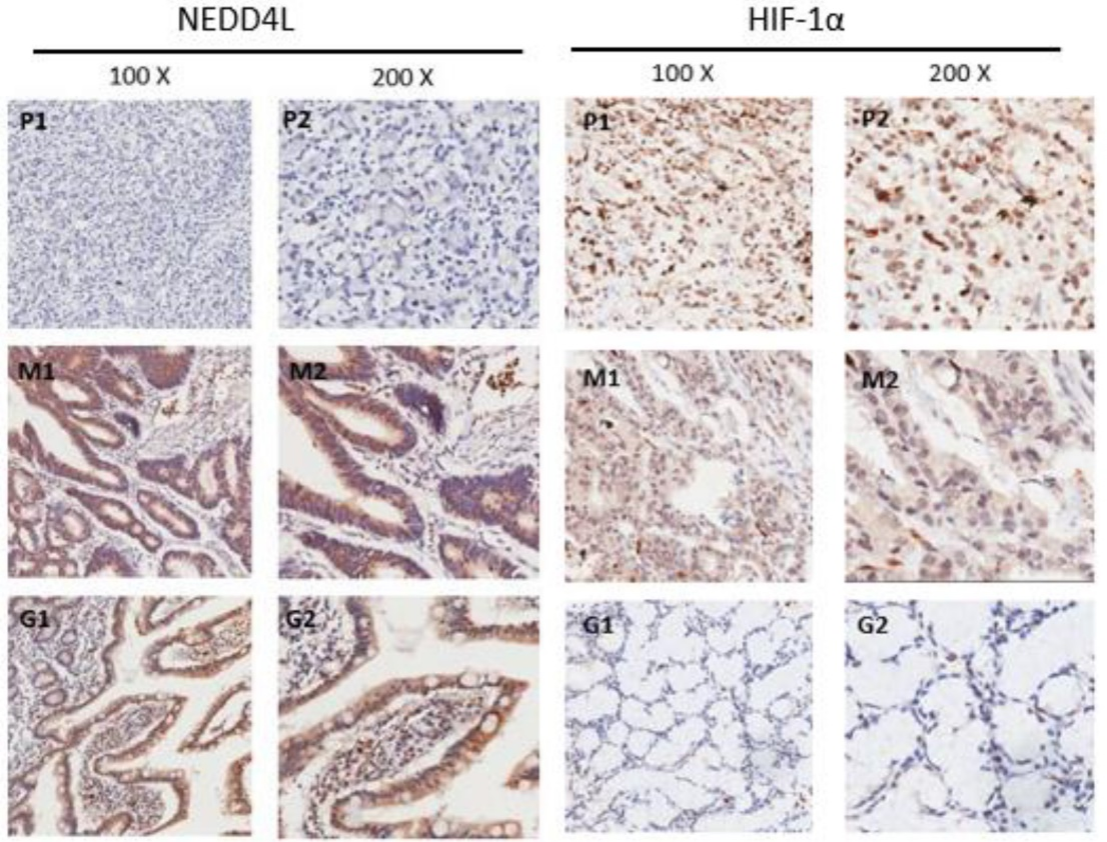
Immunohistochemical staining of NEDD4L, HIF-1α protein in GC and normal gastric tissues. Representative images of NEDD4L and HIF-1α as followings: P1, P2 (poor differentiated ) and M1, M2 (moderately differentiated) staining in GC, G1,G2 staining in normal gastric tissue. Magnification: 100× (P1, M1 and G1) and 200× (P2,M2) and G2).

To assess the NEDD4L and HIF-1α expression levels, the TMA that harbored 124 GC and 25 normal gastric tissues was detected by immunohistochemical staining. The staining results reveal the NEDD4L expression mainly in the cytoplasm and while 45.97% (57/124) in GC tissues stained positively for NEDD4L, the value was 72% (18/25) for the normal gastric tissues (P = 0.018). Moreover, while HIF-1α expressed mainly in nuclear region, and 66.13% (82/124) of gastric cancer tissues stained positive, and the value was 32% (8/25) in normal tissues (P = 0.001) (Figure 2). These results were in accordance with that of the western blot results. These results indicate that the NEDD4L expression is low in gastric cancer, whereas HIF-1α is overexpressed.

### Clinical significance and the correlation of NEDD4L and HIF-1α expression in GC

**Figure 3:**
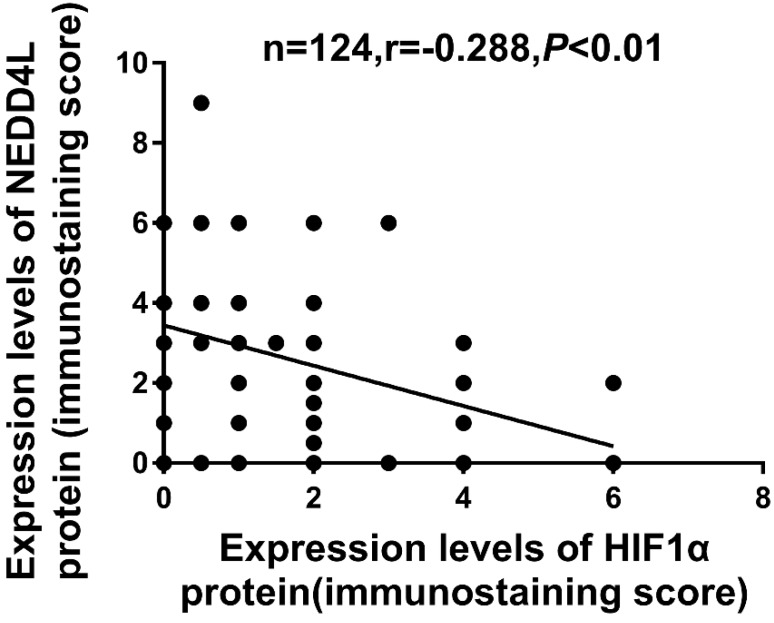
Correlation between NEDD4L and HIF-1α in GC tissues.

The Table 1 lists the clinicopathological characters of NEDD4L and HIF-1α. The low expression of NEDD4L significantly correlated with tumor invasion (P = 0.025), tumor differentiation (P = 0.039) and TNM staging (P = 0.03). Likewise, enhanced HIF-1α expression correlated with tumor size (P = 0.049), TNM staging (P = 0.014) and depth of tumor invasion (P = 0.004). Contrarily, the NEDD4L and HIF-1α expression did not correlate with gender, age, lymph node metastasis and tumor location. As shown in Figure 3 and table 3, the expression of NEDD4L in GC tissues correlated negatively with HIF-1α expression (r = -0.288, P = 0.001).

### Prognostic Significance of NEDD4L and HIF-1α in GC Patient Survival

**Figure 4:**
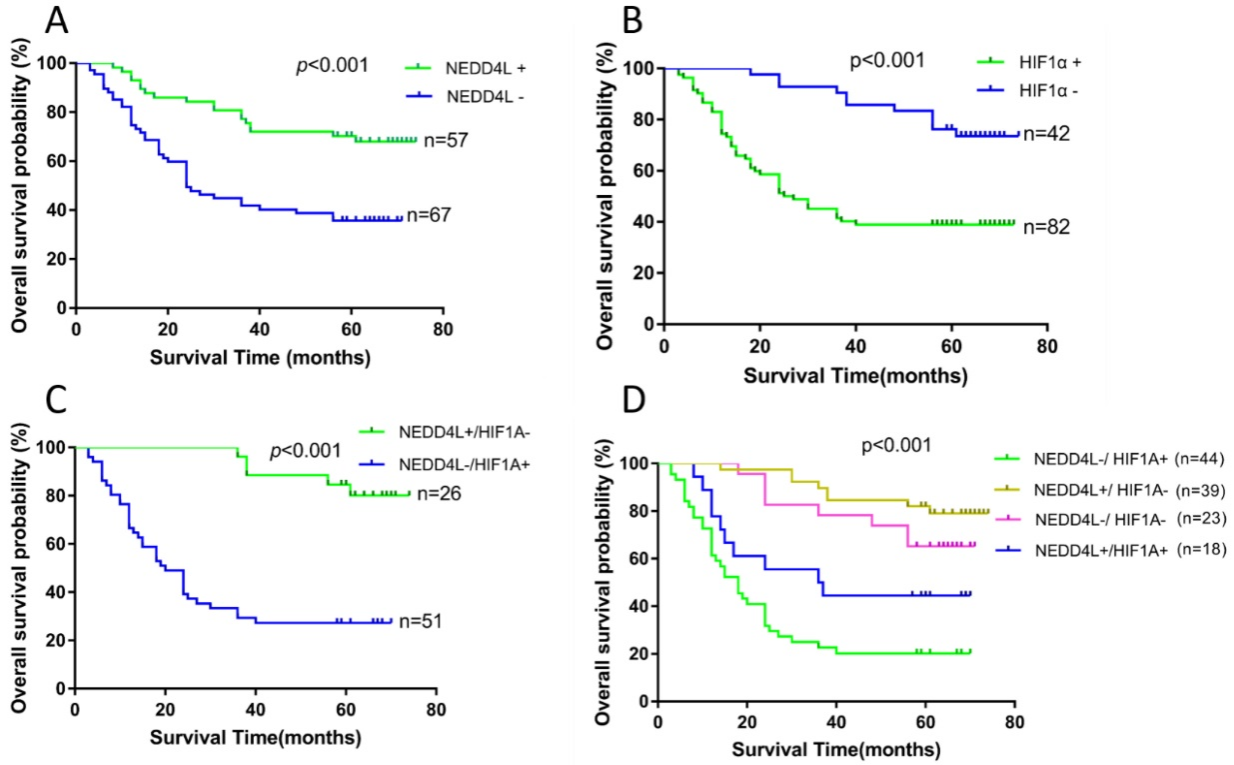
Kaplan-Meier survival analysis of the correlation between the combined NEDD4L and HIF-1α expression and the OS of GC patients. (A): The OS of patients with NEDD4L - and NEDD4L +. (B): The OS of patients with HIF1α+ and HIF-1α-. (C): Patients with NEDD4L + and HIF-1α- were compared with the patients with NEDD4L -and HIF-1α+.(D): The OS of patients with subgroups stratified by NEDD4L and HIF-1α expression.

We performed survival analysis of 124 patients using clinical follow-up results to evaluate the prognostic potential of NEDD4L and HIF-1α in GC and the results are presented in figure 4 and table 2. The patient cumulative survival rate for 3-year period with negative and positive NEDD4L expressions were 41.8% and 77.2%, respectively. Likewise, the patient cumulative survival rate for 5-year period with negative and positive expression of NEDD4L were 35.6% and 70.2%, respectively. Thus, patients with negative NEDD4L expression had a significantly worse (P < 0.001) prognosis. However, the 3- and 5- year cumulative survival rate of HIF-1α negative patients were 85.5% and 75.8%, respectively, which were notably higher than that of the survival rates of HIF-1α positive patients (30.6% and 27.2%, respectively). Apparently, high expression of HIF-1α was linked to poor prognosis for GC patients (P < 0.001).

We then explored the relationship between different combinations of NEDD4L and HIF-1α expressions and the prognosis of GC patients. Based on NEDD4L and HIF-1α expressions, the patients were divided into four groups: (1), NEDD4L-/HIF-1α+; (2), NEDD4L+/HIF-1α-; (3), NEDD4L-/HIF-1α-; (4), NEDD4L+/ HIF-1α+. Among the four groups, the worst prognosis was observed in NEDD4L-/ HIF-1α+ patients (Figure 4; mean survival time, 26.538 ± 3.530 months), whereas the best prognosis was observed in the NEDD4L+/HIF-1α- patients (mean survival time, 66.528 ± 2.550 months). While there was a significant difference between these two groups concerning OS (P < 0.001), no such difference in OS was observed between NEDD4L+/HIF-1α+ and NEDD4L-/HIF-1α- groups (p = 0.07), thus, indicating that a high level of NEDD4L or a low level of HIF-1α may functionally compensate for each other's effects in the prognosis of GC patients.

To investigate if NEDD4L and HIF-1α could independently predict the GC prognosis, we carried out Cox's univariate regression analysis and found that the parameters including tumor size, metastasis of the lymph node, depth of invasion, differentiation, TNM stage, and the NEDD4L and HIF-1α levels significantly corresponded to OS in GC patients (Table 2). Multivariate analysis revealed that for overall survival in GC patients, tumor differentiation and the combined expression of NEDD4L and HIF-1α were independent prognostic factors (Table 3).

## Discussion

The human gene NEDD4L encodes ubiquitin ligase NEDD4L, which downregulates epithelial sodium channels of kidney, which are associated with essential hypertension^22^. Subsequently, a broader role for this ubiquitin ligase has been reported in several types of tumors, with varying outcomes^3^, ^7^, ^23^. For instance, decreased NEDD4L expression corresponds to an increased prostate cancer risk, while that in NSCLC corresponds with a poor prognosis^4^.

In this study, we found that in most GC tissues the NEDD4L mRNA and protein levels were significantly reduced. This too, significantly correlated with tissue differentiation, TNM staging and depth of tumor invasion, significantly shorter survival, as was seen in the subsequent survival analysis, and is in accordance with previous reports. Multivariate Cox analysis revealed that NEDD4L was an independent predictor factor of GC.

NEDD4L affects tumor-associated pathways through ubiquitination and plays an important role in tumorigenesis and development. For example, Kuratomi et al and Gao et al. found that NEDD4L inhibited the TGF-β signaling pathway by accelerating the ubiquitination of activated Smads and promoting their degradation^24^, ^25^. NEDD4L also target Dvl2, a key component of Wnt signaling, and negatively regulate Wnt signaling pathways^26^. LoïcBroix et al. reported that NEDD4L dysregulates the AKT-mTOR pathway by disrupting mTORC1-mediated signaling, a finding similar to another report showing that NEDD4L catalyzed the PIK3CA ubiquitination and regulated PI3K-AKT signaling^21^. Therefore, NEDD4L may be considered a tumor suppressor, although, this needs to be verified through direct and robust experimental evidence. Our previous experimental results indicate that NEDD4L is associated with tumor differentiation, invasion, and metastasis, and NEDD4L expression is also associated with the prognosis of patients with gastric cancer.

The tumor microenvironment is characterized by nutrient supply diversity, pH, and oxygenation^27^. Particularly, hypoxia is associated with tumor development, progression, metastasis, inadequate response to treatment, and changes in tumor cell behavior through many O^2^-sensitive pathways. The most studied is HIFs mediation^28^, ^29^. This NEDD4L ubiquitination is affected by hypoxic stress in tumor tissues; therefore, we hypothesized that NEDD4L might be associated with hypoxia-inducible gene HIF-1α.

HIF-1α mRNA and protein levels were significantly higher in tumor tissues than in normal tissues. The survival analysis also revealed a notably shorter survival time for GC patients with high HIF-1α levels than those with lower expression. Furthermore, increased HIF-1α expression also correlated with tumor size, TNM staging, and depth of tumor invasion. These results corroborated with the multivariate Cox regression analysis and reveal that HIF-1α is a factor for the GC prognosis. Our results indicate that the role of HIF-1α in gastric cancer is the opposite of NEDD4L.

Given the correlation between NEDD4L and HIF-1α, we hypothesized that they may be jointly involved in tumor development and progression. We then explored the link between the expressions of NEDD4L and HIF-1α in GC and ultimately their relationship with patient prognosis. Our results showed an inverse relationship between NEDD4L and HIF-1α expressions (r = -0.288, P < 0.01). Additionally, we observed the worst prognosis in the NEDD4L- / HIF-1α+ patients, suggesting that the correlation of NEDD4L and HIF-1α is a more credible indicator of GC prognosis, although, this requires further study to determine the specific mechanism involved.

Thus, our results indicate that NEDD4L and HIF-1α may be independent prognostic factors for GC. Concurrently, a more important prognostic indicator of GC patients may be the combination of NEDD4L and HIF-1α. Therefore, the mechanisms that NEDD4L- and HIF-1α- mediated regulation of GC progression need to be explored further, which may ultimately promote the development of new anti-cancer strategies.

## Figures and Tables

**Table 1:** Relationship between NEDD4L and HIF-1α expression and clinicopathological variables (n=124)

Variables	Total	NEDD4L expression		*P* value	HIF-1α expression		*P* value
		negative	positive		negative	positive	
**Gender**				0.670			0.826
Male	96	43	53		33	63	
Female	28	14	14		9	19	
**Age at surgery (y)**				1			0.312
<61	61	33	28		18	43	
≥61	63	29	34		24	39	
**Tumor size (cm)**				0.448			**0.049**
<6	41	42	40		9	32	
≥6	83	25	17		33	50	
**Differentiation**				**0.039**			0.349
High/moderate	32	12	20		13	19	
poor/no	92	55	37		29	63	
**Location**				0.975			0.854
Cardia	64	34	30		22	42	
Corpus	27	15	12		8	19	
antrum	33	18	15		12	21	
**T stage**				**0.025**			**0.004**
T1/T2	26	9	17		15	11	
T3/T4	98	58	40		27	71	
**N stage**				0.448			0.097
No	41	47	36		18	23	
Yes	83	20	21		24	59	
**TNM**				**0.030**			**0.014**
I/II	52	30	22		24	28	
III/IV	72	45	27		18	54	

**Table 2:** Univariate analysis of the correlation between clinicopathological factors and survival of patients with GC

Parameters	Cumulative Survival Rates, %		Mean Survival Time, month	*P* value
	3-Year	5-Year		
**Gender**				0.452
Male	63.5	47.8	47.4	
Female	64.3	60.3	49.5	
**Age(y)**				0.633
<61	62.3	53.9	48.3	
≥61	60.3	49.2	47	
**Tumor diameter (cm)**				0.016
<6	46.3	39	39.5	
≥6	67.5	57.8	51.9	
**Location**				0.148
Cardia	62.5	46.6	47.1	
Corpus	48.1	44.4	40.8	
antrum	66.7	66.7	54.3	
**Depth of invasion**				0.000
T1/T2	96.2	92.3	68.2	
T3/T4	48	40.7	42.1	
**Histological grade**				0.001
Well/moderate	81.3	78	62	
Poor/not	50	42.4	43	
**Lymph node metastasis**				0.000
No	87.8	82.9	66.8	
Yes	43.4	34.6	38.3	
**TNM stage**				0.000
I-II	88.5	84.6	67.1	
III-IV	36.1	27.4	33.9	
**NEDD4L expression**				0.000
negative	41.8	35.6	37.7	
positive	77.2	70.2	59.1	
**HIF1α expression**				
negative	85.5	75.8	64.4	0.000
positive	30.6	27.2	30.8	
**NEDD4L/HIF1α expression**				0.000
NEDD4L-/HIF1α-	78.3	65.2	58.8	0.000
NEDD4L+/HIF1α-	89.7	82.1	66.5	
NEDD4L-/HIF1α+	22.7	20.2	26.5	
NEDD4L+/HIF1α+	50	44.4	41.4	
NEDD4L+/HIF1α-	89.7	82.1	66.5	0.000
NEDD4L-/HIF1α+	22.7	20.2	26.5	
NEDD4L-/HIF1α-	78.3	65.2	58.8	0.070
NEDD4L+/HIF1α+	50	44.4	41.4	

**Table 3:** Univariate and multivariate analysis of the correlation between clinicopathological factors and prognosis

Variables	Univariate analysis	*P* value	Multivariate analysis	*P* value
	HR(95%CI)		HR(95%CI)	
Gender (male vs. female)	0.782(0.407-1.503)	0.46		NI
Age (y) (<61 vs. ≥61)	1.128(0.682-1.864)	0.639		NI
Tumor size (cm) (<6 vs. ≥6)	0.544(0.326-0.905)	**0.019**	1.140(0.670-1.939)	0.63
Histological grade (Poor and other vs Well and moderate)	3.425(1.557-7.535)	**0.002**	2.715(1.224-6.023)	**0.014**
Depth of invasion (T3/T4 vs T1/T2)	11.393(2.779-46.7)	**0.001**	2.674(0.393-18.209)	0.315
Lymph node metastasis (yes vs. no)	5.771(2.619-12.715)	**1.37E-05**	2.539(0.200-32.213)	0.472
TNM stages ( III/IV vs I/II.)	7.987(3.779-16.880)	**5.26E-08**	2.282(0.165-31.455)	0.583
NEDD4L expression (+ VS- )	0.354(0.204-0.615)	**2.30E-4**	0.494(0.279-0.875)	**0.016**
HIF1αexpression(+ vs -)	5.196(2.913-9.268)	**2.38E-8**	4.606(2.450-8.661)	**2.12E-06**
NEDD4L/HIF1α expression (NEDD4L-/HIF1α+ VS NEDD4L+/HIF1α-)	0.121(0.055-0.266)	**1.36E-07**	0.140(0.059-0.333)	**4.42E-07**
